# A novel small-molecule arylsulfonamide causes energetic stress and suppresses breast and lung tumor growth and metastasis

**DOI:** 10.18632/oncotarget.22104

**Published:** 2017-10-29

**Authors:** Xin Dai, Stefan Kaluz, Ying Jiang, Lei Shi, DeAngelo Mckinley, Yingzhe Wang, Binghe Wang, Erwin G. Van Meir, Chalet Tan

**Affiliations:** ^1^ Department of Pharmaceutics and Drug Delivery, University of Mississippi, Oxford, MS, USA; ^2^ Department of Neurosurgery, Emory University, Atlanta, GA, USA; ^3^ Department of Hematology and Medical Oncology, Emory University, Atlanta, GA, USA; ^4^ Winship Cancer Institute, Emory University, Atlanta, GA, USA; ^5^ Department of Pharmaceutical Sciences, Mercer University, Atlanta, GA, USA; ^6^ Department of Biology, Georgia State University, Atlanta, GA, USA; ^7^ Department of Chemistry, Georgia State University, Atlanta, GA, USA

**Keywords:** arylsulfonamide, AMPK, mTORC1, HIF-1, metastasis

## Abstract

Neoplastic cells display reprogrammed metabolism due to the heightened energetic demands and the need for biomass synthesis of a growing tumor. Targeting metabolic vulnerabilities is thus an important goal for cancer therapy. Here, we describe a novel small-molecule arylsulfonamide (N-cyclobutyl-N-((2,2-dimethyl-2*H*-pyrano[3,2-*b*]pyridin-6-yl)methyl)-3,4-dimethoxybenzenesulfonamide) that exerts potent cytotoxicity and energetic stress on tumor cells while largely sparing non-cancerous human cells. In tumor cells, it stimulates glycolysis and accelerates glucose consumption. Consequently, intracellular ATP levels plummet, triggering activation of AMP-activated protein kinase (AMPK), and diminishing the mammalian target of rapamycin complex 1 (mTORC1) and hypoxia-inducible factor 1 (HIF-1) signaling. In orthotopic triple-negative breast cancer and subcutaneous lung cancer mouse models, this arylsulfonamide robustly suppresses primary tumor growth, inhibits the formation of distant metastases to the lung, and extends mouse survival while being very well tolerated. These therapeutic effects are further potentiated by co-administration of 2-deoxy-D-glucose (2-DG), a glucose analog and glycolysis inhibitor. Collectively, our findings provide preclinical proof of concept for the further development of this arylsulfonamide in combination with 2-DG towards cancer treatment.

## INTRODUCTION

Uncontrolled tumor cell proliferation and growth impose tremendous demand for ATP, amino acids, lipids and nucleotides. To fuel the anabolic processes, tumor cells consume a much larger quantity of glucose than their normal counterparts and metabolize it predominantly through glycolysis even under oxygen-rich conditions, the metabolic phenotype commonly referred to as aerobic glycolysis or Warburg effect [1, 2]. In normal differentiated mammalian cells, glucose is catabolized to CO_2_ in a stepwise fashion via glycolysis, mitochondrial tricarboxylic acid (TCA, Krebs) cycle and oxidative phosphorylation (OXPHOS). In contrast, in tumor cells, glucose primarily undergoes glycolysis as mitochondrial oxidation is greatly reduced. Although the majority of glucose-derived carbon is converted to lactate and subsequently excreted into the extracellular space, substantial amounts of metabolic intermediaries are generated to serve as essential precursors for macromolecular biosynthesis [3].

Glycolysis occurs in the cytosol and is inefficient in terms of ATP production, as it yields only two ATP molecules per molecule of glucose, whereas the complete oxidation of each glucose molecule in mitochondria can generate up to 34 ATP molecules. A careful evaluation of the energy budget reveals that glycolysis is not the dominant producer of ATP in most tumor cell types [4, 5]. In fact, about 5% of the glucose flux still enters the mitochondria, and oxidation of metabolic intermediaries through the TCA cycle and OXPHOS contributes to about half of the total ATP production in tumor cells [1].

AMP-activated protein kinase (AMPK) is a ubiquitous energy sensor in eukaryotes that is responsive to changes in intracellular AMP/ATP ratio. AMPK acts as a metabolic checkpoint and a key regulator of energy homeostasis by promoting catabolic pathways generating ATP while switching off ATP-consuming anabolic processes, such as protein synthesis [6, 7]. Pivotal among the downstream targets of AMPK is the mammalian target of rapamycin complex 1 (mTORC1), a serine/threonine protein kinase highly conserved in all eukaryotes that coordinates cell growth with energy and nutrient availability [8]. A number of oncogenic events, such as inactivating mutations of tumor suppressor genes and hyperactivation of oncogenes converge on mTORC1 upregulation. Attenuating mTORC1 kinase activity thus represents an important objective in cancer therapy as this halts cell growth [9].

Hypoxia is a hallmark of the solid tumor microenvironment. The partial oxygen tension in normal tissues ranges from 30–60 mmHg, whereas in human tumors it is mostly below 10 mmHg (equivalent to 1.4% O_2_) or even less than 5 mmHg [10, 11]. Intratumoral hypoxia has long been associated with malignant phenotypes, poor prognosis and resistance to radiotherapy and chemotherapy [12]. The main mediators of biological responses to hypoxic stress in mammalian cells are hypoxia-inducible factors (HIF-1 and HIF-2), heterodimeric transcription factors composed of an oxygen-labile α–subunit (HIF-1α or HIF-2α) and a stable β-subunit (HIF-1β). By transcriptionally controlling the expression of over 100 target genes, HIFs are critically involved in tumor initiation, metabolic adaptation to hypoxia, apoptotic resistance, cancer stem cell growth, inflammatory cell recruitment, angiogenesis and metastasis [13, 14].

We have previously synthesized and characterized a series of small-molecule arylsulfonamides for their ability to block hypoxia/HIF-mediated transcription and related anticancer effects [15–20]. Structure-activity relationship optimization studies led us to develop novel arylsulfonamide N-cyclobutyl-N-((2,2-dimethyl-2*H*-pyrano[3,2-*b*]pyridin-6-yl)methyl)-3,4-dimethoxybenzenesulfonamide (abbreviated as 64B, Supplementary Figure 1) as a promising new lead compound with augmented anti-HIF potency and improved solubility [15]. Here, we report for the first time on the metabolic perturbation and anticancer efficacy of this optimized arylsulfonamide. We demonstrate that 64B alters glucose metabolism and depletes intracellular ATP, resulting in AMPK activation and diminished mTORC1/HIF-1 signaling in tumor cells. Furthermore, 64B strongly suppresses tumor growth and metastasis in triple-negative breast and lung cancer mouse models, which is further potentiated by co-administration with glycolysis inhibitor 2-deoxy-D-glucose (2-DG), supporting its further development towards cancer treatment.

## RESULTS

### 64B exerts potent cytotoxicity in tumor cells and impairs their motility

We first evaluated the cytotoxicity of 64B in a variety of human and murine tumor cell lines harboring various oncogenic mutations. The IC_50_ of 64B was found to be in the low micromolar range in tumor cells originating from breast, lung, pancreas, skin (melanoma) and brain (glioblastoma). By contrast, 64B showed little cytotoxicity in non-cancerous breast epithelial MCF-10A and lung fibroblast MRC-5 cells (Table 1). These results provide compelling evidence that 64B is cytotoxic against tumor cells independent of tissue origin or oncogenic mutations.

**Table 1:** Cytotoxicity of 64B in tumor cell lines with various tissue origins and oncogenic mutations. All cells are human except where indicated.

Cell lines	IC_50_ (μM)	Tissue	Mutated Genes
MCF-7	0.15	Breast cancer	*PIK3CA*
MDA-MB-231	1.8	Breast cancer	*KRAS*, *TP53*, *BRAF*
A549	2.2	Lung cancer	*KRAS*
NCI-H1975	2.7	Lung cancer	*TP53*, *PIK3CA*, *EGFR*
LLC	3.0	Lung cancer (murine)	--
BxPC-3	1.9	Pancreatic cancer	*TP53*
Panc-1	2.6	Pancreatic cancer	*KRAS*, *TP53*
SK-MEL-28	5.6	Melanoma	*BRAF*, *TP53*, *CDK4*
B16-F10	4.0	Melanoma (murine)	--
LN229	3.2	Glioblastoma	*ERBB2*
MRC-5	>20	Lung fibroblast cells	--
MCF-10A	>20	Breast epithelial cells	--

**Figure 1:**
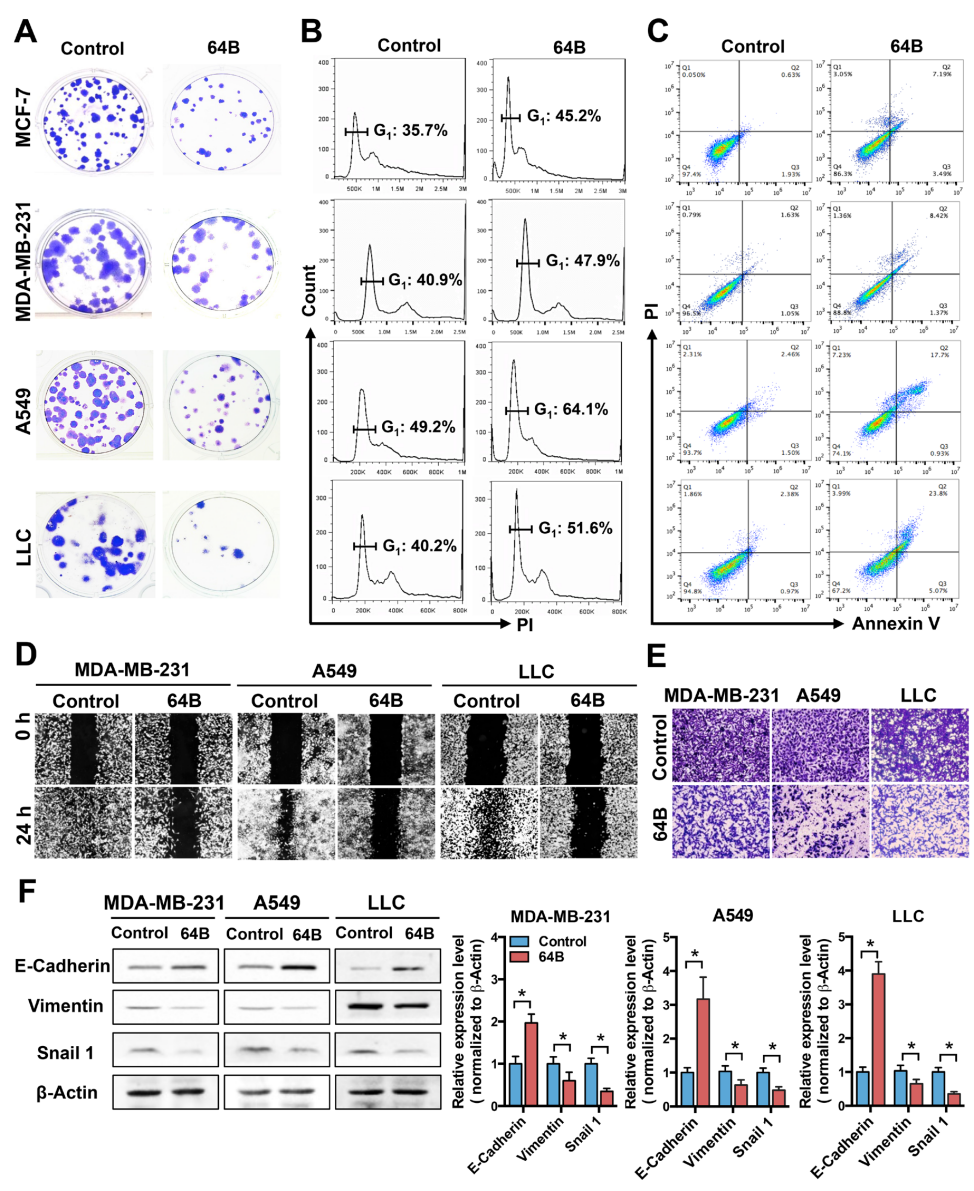
64B exerts potent cytotoxicity in tumor cells and impairs their motility MCF-7, MDA-MB-231, A549 and LLC cells were pre-treated with 64B (5 μM) for 24 h prior to starting the assays below. **A**, 64B reduces colony formation of tumor cells. Colonies were visualized by crystal violet staining following 14-day incubation. **B**, 64B causes G1 arrest in tumor cells. Cells were stained with propidium iodide (PI) and the DNA content was analyzed by flow cytometry, the cell cycle distribution was quantified by FlowJo software. **C**, 64B induces apoptosis in tumor cells. Following dual staining with Annexin V-FITC and PI, cells were analyzed by flow cytometry. **D**, 64B impedes tumor cell migration. Scratch wounds were generated in cell monolayer and the amount of gap closure measured following 24-h incubation. **E**, 64B diminishes tumor cell invasion. Cells were seeded in matrigel-coated transwell chambers and incubated for 24 h, and the invading cells on the bottom of the chamber inserts were stained with crystal violet. **F**, Western blot analysis showing the protein levels of E-cadherin, vimentin, and Snail1 in 64B-treated tumor cells. Protein band intensities were quantified by densitometric analysis using ImageJ software. All data show representative results obtained from three independent experiments, and the results are reported as the mean ± SD (n = 3). *, *p* < 0.05.

In colony formation assays, the number and size of colonies formed by 64B-pretreated tumor cells were clearly reduced compared to controls (Figure 1A), while those of MCF-10A and MRC-5 cells were minimally affected (Supplementary Figure 2). Cell cycle analysis showed that 64B significantly increased the G1 phase in all tested tumor cell lines (Figure 1B). Moreover, 64B increased the cell population undergoing late apoptosis (Annexin V^+^/PI^+^) by 5–10 fold (Figure 1C). These results indicate that 64B inhibits tumor cell proliferation via G1 arrest and induction of apoptosis.

Epithelial cancer cells are well known for their high motility, which is attributed to the process of epithelial-to-mesenchymal transition (EMT) [21]. In scratch wound healing cell migration assays, tumor cells in the control group showed nearly complete wound closure as illustrated by the gap area being largely filled with migrating tumor cells, whereas the treatment with 64B severely blocked wound closure (Figure 1D). In transwell matrigel invasion assays, 64B notably reduced the invading cell population compared to controls (Figure 1E). Consistent with the less motile phenotype, the protein level of epithelial cell marker E-cadherin was significantly elevated in 64B-treated tumor cells, accompanied by a reduction in vimentin and Snail1, two mesenchymal markers (Figure 1F). These results indicate that 64B impairs the motility of tumor cells.

### 64B alters glucose metabolism and depletes ATP in tumor cells

**Figure 2:**
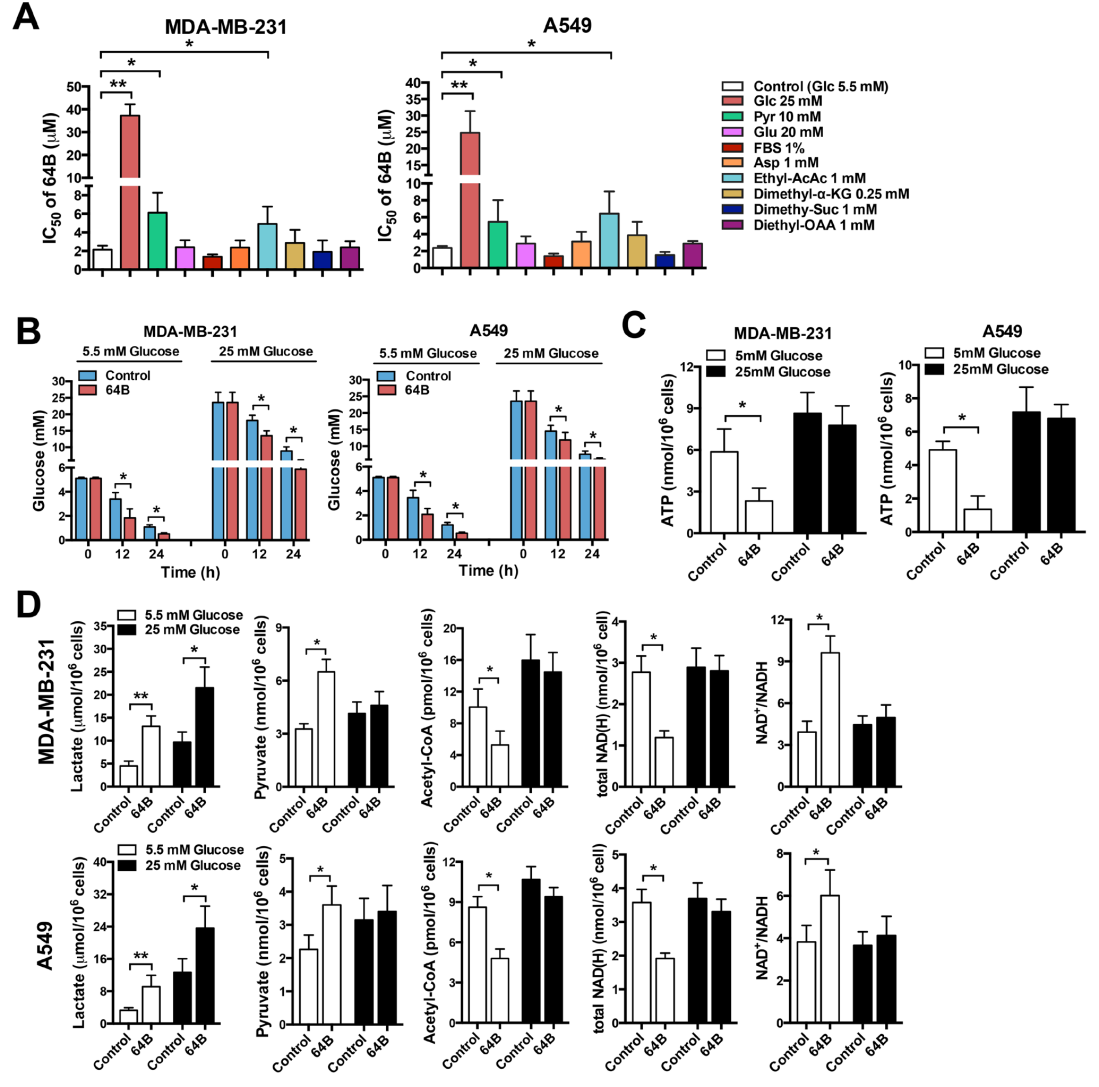
64B alters glucose metabolism and depletes ATP in tumor cells Tumor cells were cultured in normoglycemic or hyperglycemic DMEM, and were treated with 64B (5 μM) for 24 h unless indicated otherwise. **A**, Cell proliferation assay showing the IC_50_ of 64B in MDA-MB-231 and A549 cells cultured for 72 h in the presence of 5.5 mM or 25 mM glucose, 1 mM or 10 mM pyruvate, 2 mM or 20 mM glutamine, 0.1 mM or 1 mM aspartate, 1% FBS or 10% FBS, 1 mM ethyl acetoacetate, 0.25 mM dimethyl α-KG, 1 mM dimethyl succinate and 1 mM diethyl OAA. **B**, Effect of 64B on glucose consumption in tumor cells by measuring the glucose level in culture medium. **C**, Effect of 64B on intracellular ATP level. **D**, Effect of 64B on the levels of metabolic intermediaries in tumor cells. All data show representative results obtained from three independent experiments, and the results are reported as the mean ± SD (n = 3). *, *p* < 0.05. **, *p* < 0.01.

To determine whether 64B cytotoxicity is related to metabolic vulnerabilities in tumor cells, we examined whether various nutrients (glucose, pyruvate, glutamine, aspartate, serum) could rescue the cytotoxicity of 64B. Raising the glucose content in culture medium from 5.5 mM (1 g/L, physiological level, normoglycemia) to 25 mM (4.5 g/L, hyperglycemia) drastically weakened the cytotoxicity of 64B in tumor cells as evidenced by over 10-fold increase in its IC_50_, implying the involvement of glucose metabolism in 64B action (Figure 2A). Elevating pyruvate concentration in the culture medium also compromised the cytotoxicity of 64B as reflected by the doubled IC_50_. On the other hand, an increase in the level of glutamine or aspartate barely had any effect (Figure 2A), indicating that glutamine or aspartate anaplerosis cannot reverse 64B action. To further study the effect of TCA-cycle intermediaries on 64B activity, we employed the lipophilic precursors ethyl acetoacetate, dimethyl α-ketoglutarate (α-KG), dimethyl succinate and diethyl oxaloacetate (OAA) given their improved cell membrane permeability, as the alkyl groups can be hydrolyzed by intracellular esterases [22]. Although replenishing α-KG, succinate or OAA had negligible effects, the addition of acetoacetate, a precursor of acetyl-CoA, partially rescued the cytotoxicity of 64B (Figure 2A). As pyruvate is known to be oxidized to form acetyl-CoA, the commitment step into the TCA cycle [3], these results suggest a blockade in the conversion of pyruvate to acetyl-CoA in 64B-treated tumor cells.

To further investigate the link between 64B activity and altered glucose metabolism, we examined glucose consumption and energetic status in tumor cells. 64B-treated tumor cells consumed significantly more glucose than controls under both normo- and hyper-glycemic conditions, as reflected by a steeper decline in glucose levels in culture medium over a 24-h incubation period (Figure 2B). In tumor cells cultured under normoglycemia, 64B markedly reduced intracellular ATP levels within 12 h of incubation, which was further exacerbated by 24 h (Figure 2C and Supplementary Figure 3A). Such effect was absent in tumor cells grown in hyperglycemic medium. These results indicate that 64B causes intracellular ATP depletion in a glucose-dependent manner while accelerating glucose consumption.

To corroborate 64B-mediated perturbation of glucose metabolism in tumor cells, we next assessed the levels of key glucose metabolites. In tumor cells, even in the presence of abundant O_2_, glucose is sequentially catabolized to form pyruvate, which is predominantly reduced in the cytosol to generate lactate and NAD^+^, the latter serving as an important electron acceptor in both glycolysis and the TCA cycle. A small fraction of the pyruvate flux enters the mitochondria and is irreversibly oxidized to form acetyl-CoA, the commitment step into the TCA cycle [3]. We found that 64B-treated tumor cells secreted significantly higher levels of lactate into the culture medium than the controls, indicating that 64B stimulates aerobic glycolysis (Figure 2D and Supplementary Figure 3B). In tumor cells grown under normoglycemic conditions, 64B caused a significant increase in pyruvate along with a sharp decline in acetyl-CoA. The total cellular NAD(H) level was markedly decreased and accompanied by an increased NAD^+^/NADH ratio, reflecting a drastic decline in intracellular NADH level. The buildup of intracellular pyruvate and the concomitant reduction in acetyl-CoA and NADH is consistent with the notion that 64B treatment results in decreased entrance of pyruvate into the TCA cycle, which diminishes ATP production in the mitochondria. Of note, 64B caused negligible metabolic disturbance and energetic stress under hyperglycemia (Figure 2C, 2D), in agreement with weakened 64B cytotoxicity observed in Figure 2A.

### 64B downregulates mTORC1 signaling and disrupts HIF-1 transactivation

**Figure 3:**
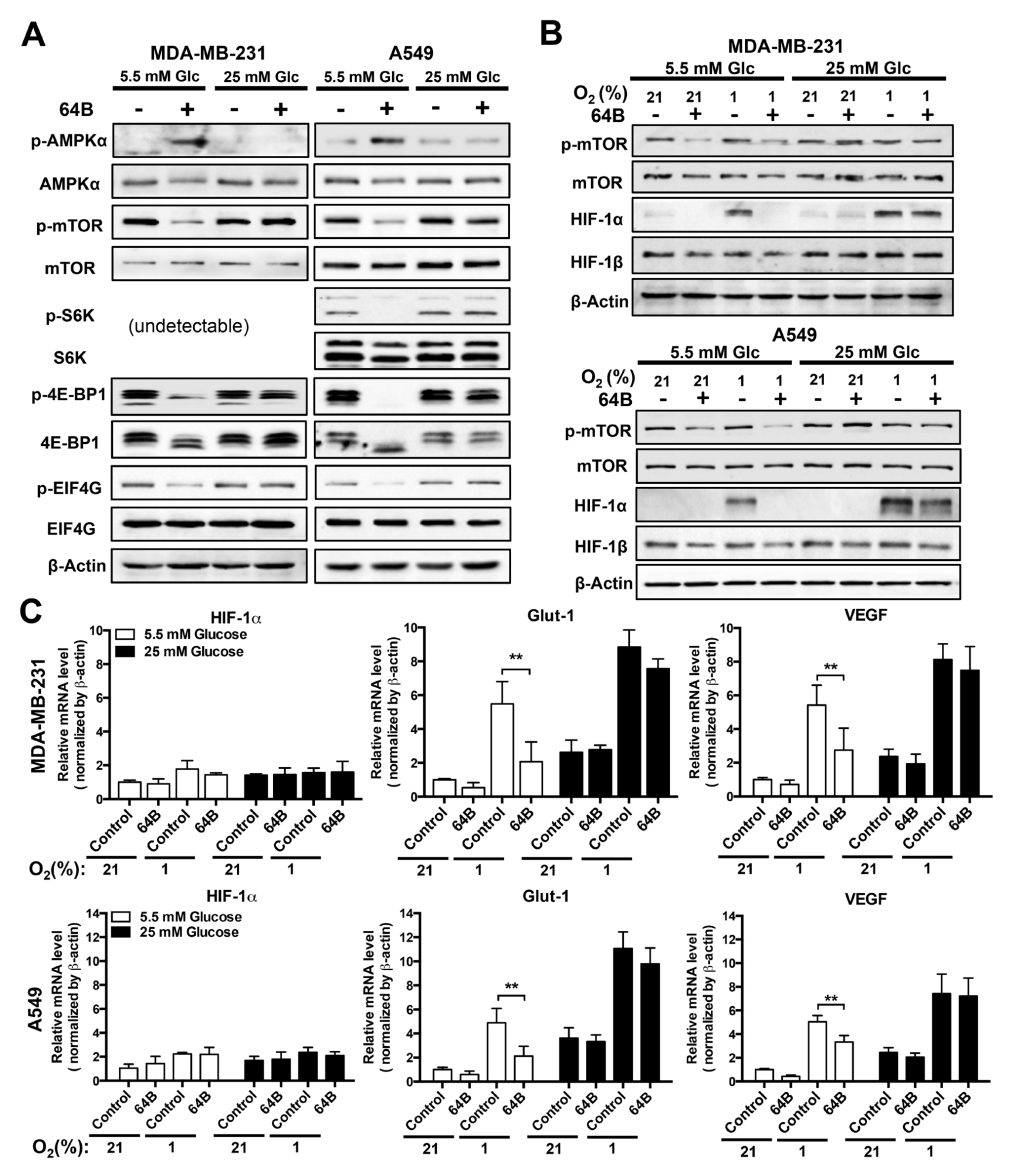
64B downregulates mTORC1 signaling and disrupts HIF-1 transactivation in tumor cells Tumor cells were cultured in normoglycemic or hyperglycemic DMEM under normoxia (21% O_2_) or hypoxia (1% O_2_), and were treated with 64B (5 μM) for 24 h. A,B, Western blot analysis showing the effect of 64B on the levels of indicated proteins under normoxia (**A**) and hypoxia (**B**). **C**, qRT-PCR measuring the expression of *HIF-1α*, *Glut-1* and *VEGF* mRNAs in 64B-treated tumor cells under hypoxia. All data show representative results obtained from three independent experiments, and the results are reported as the mean ± SD (n = 3). *, *p* < 0.05. **, *p* < 0.01.

Depletion of intracellular ATP is known to activate AMPK, which in turn inhibits mTORC1 signaling, leading to global shutdown of protein synthesis [6]. We found strong p-AMPK induction 24 h after tumor cell treatment with 64B under normoglycemia, which was accompanied with a decrease in p-mTOR1 and its downstream effectors S6 ribosomal protein kinase 1 (S6K1), 4E-BP1 and EIF4G (Figure 3A). These effects were absent under hyperglycemic conditions.

To gauge the effect of 64B on mTORC1-dependent protein translation, we then examined the protein level of HIF-1α, the oxygen-sensitive and regulatory subunit of HIF-1, which is also a sensor of translation efficiency due to its rapid turnover [23]. In tumor cells grown in normoglycemic medium, 64B abrogated hypoxia (1% O_2_)-induced HIF-1α protein accumulation, whereas HIF-1β remained unaltered (Figure 3B). Consequently, this led to attenuated transactivation of HIF-1 target genes under hypoxia, as exemplified by reduced *glut-1* and *vegf* transcription in 64B-treated tumor cells without affecting *HIF-1α* mRNA level (Figure 3C). Such effects were absent under hyperglycemic conditions. Together, these results demonstrate that 64B triggers the energy sensor AMPK in tumor cells and downregulates mTORC1 signaling, leading to diminished HIF-1α protein synthesis, in effect disrupting the hypoxia-induced HIF-1 transcriptome.

### Glucose deprivation potentiates the anticancer activity of 64B

**Figure 4:**
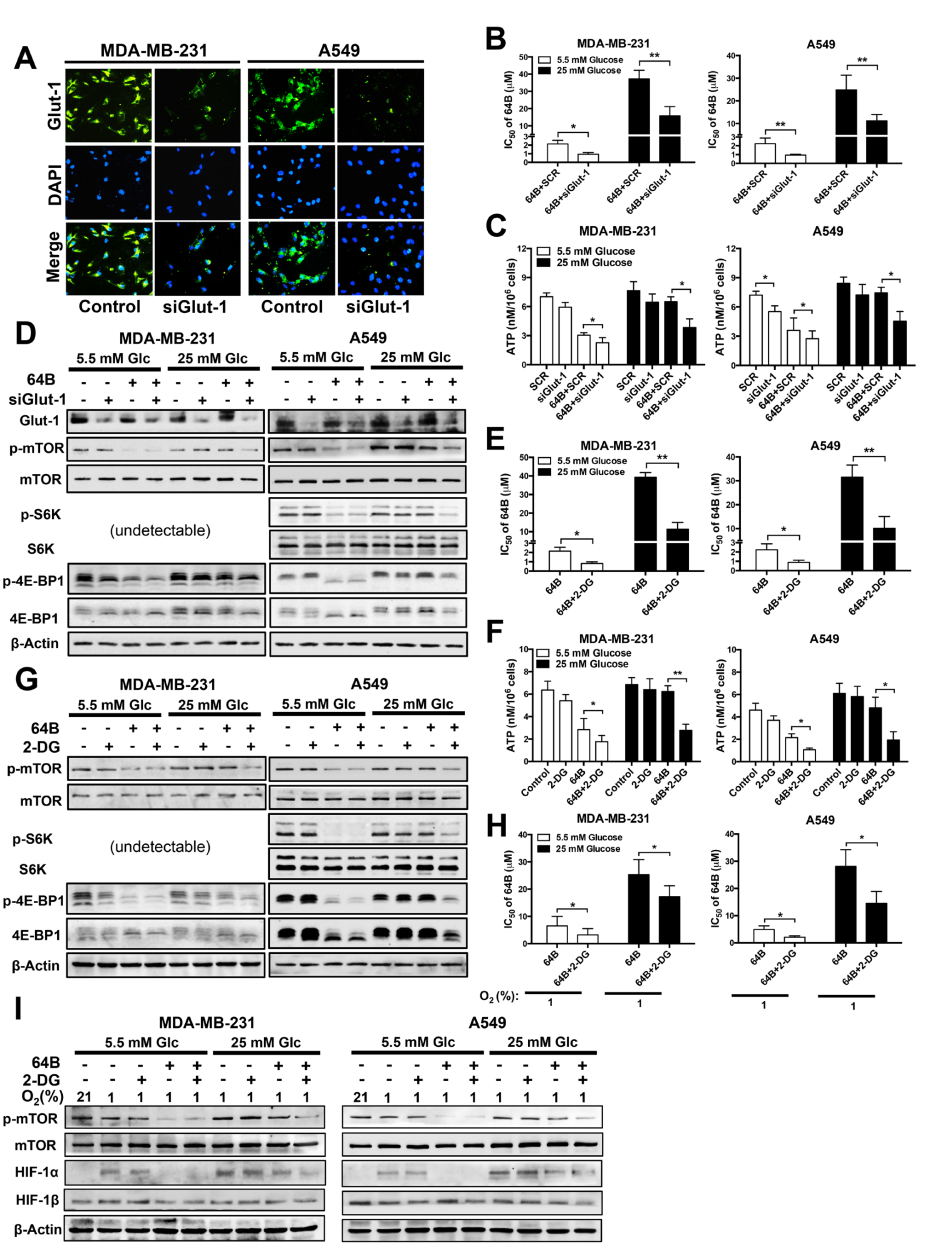
Glucose deprivation potentiates the anticancer activity of 64B MDA-MB-231 and A549 cells were cultured in normoglycemic or hyperglycemic DMEM, and were treated with 64B (5 μM) and/or 2-DG (2 mM) for 24 h unless indicated otherwise. **A**, Knockdown of Glut-1 expression by si*Glut-1* was verified by immunofluorescent staining in tumor cells after 48 h. The nuclei of tumor cells were counter-stained with DAPI. **B**, **C**, Effect of the co-treatment with si*Glut-1* and 64B on the cytotoxicity IC_50_ of 64B in tumor cells (**B**) and intracellular ATP level (**C**). Equal amount of scrambled siRNA (SCR) was used as a control. **D**, Western blot analysis showing effect of co-treatment with si*Glut-1* and 64B on levels of indicated proteins. **E**, **F**, Effect of co-treatment with 64B and 2-DG on the cytotoxicity IC_50_ of 64B in tumor cells (**E**) and intracellular ATP level (**F**). **G**, Western blot analysis showing the effect of combined treatment with 64B and 2-DG on levels of indicated proteins. **H**, Effect of co-treatment with 64B and 2-DG on the cytotoxicity IC_50_ of 64B under hypoxia (1% O_2_) for 72 h. **I**, Western blot analysis showing effect of co-reatment with 64B and 2-DG on levels of indicated proteins under hypoxia. All data show representative results obtained from three independent experiments, and the results are reported as the mean ± SD (n = 3). *, *p* < 0.05. **, *p* < 0.01.

Given the inverse correlation between glucose abundance and 64B activity (Figures 2 and 3), we hypothesized that glucose deprivation would sensitize tumor cells to the cytotoxicity of 64B. We took two approaches to block glucose utilization in tumor cells. First, we reduced glucose availability by knockdown of *glut-1* using siRNA. Glut-1 is the primary transporter that mediates glucose uptake into human epithelial cells [24]. We found that *glut-1* siRNA greatly reduced Glut-1 protein expression (Figure 4A, 4D) and enhanced the cytotoxicity of 64B in tumor cells by 2-fold (Figure 4B), which resulted in a more severe reduction in intracellular ATP level (Figure 4C) and mTORC1 signaling (Figure 4D). Notably, co-treatment of *glut-1* siRNA with 64B was able to sensitize hyperglycemic tumor cells to its cytotoxic effects.

Second, we used 2-DG, a glucose analog that competes with glucose uptake and inhibits glycolytic enzyme activity [25]. Again, 2-DG pronouncedly sensitized tumor cells to the cytotoxicity of 64B, and its effect was accentuated under hyperglycemia (Figure 4E). Importantly, in hyperglycemic tumor cells, the combined treatment with 64B and 2-DG significantly reduced intracellular ATP (Figure 4F) and mTORC1 signaling (Figure 4G), which was not achieved by either agent individually. Similarly, the sensitization effects of 2-DG on 64B activity were also observed in tumor cells under hypoxia (Figure 4H, 4I). Together, these results clearly indicate that glucose deprivation enhances the cytotoxicity of 64B in tumor cells.

### Combined treatment with 64B and 2-DG potently suppresses tumor growth and metastasis

We next investigated the therapeutic potential of 64B/2-DG combination *in vivo*. We first characterized the pharmacokinetic profile of 64B in mice bearing orthotopic MDA-MB-231 breast tumor xenografts. 64B was readily and continuously absorbed from the intraperitoneal cavity, as reflected by the sustained plasma concentration of about 3 µM observed between 15–60 min following i.p. injection (Supplementary Figure 4A). Co-administration of 2-DG minimally influenced the levels of 64B in plasma, tumor, brain and liver (Supplementary Figure 4B), indicating that 2-DG does not interfere with the pharmacokinetics of 64B.

**Figure 5:**
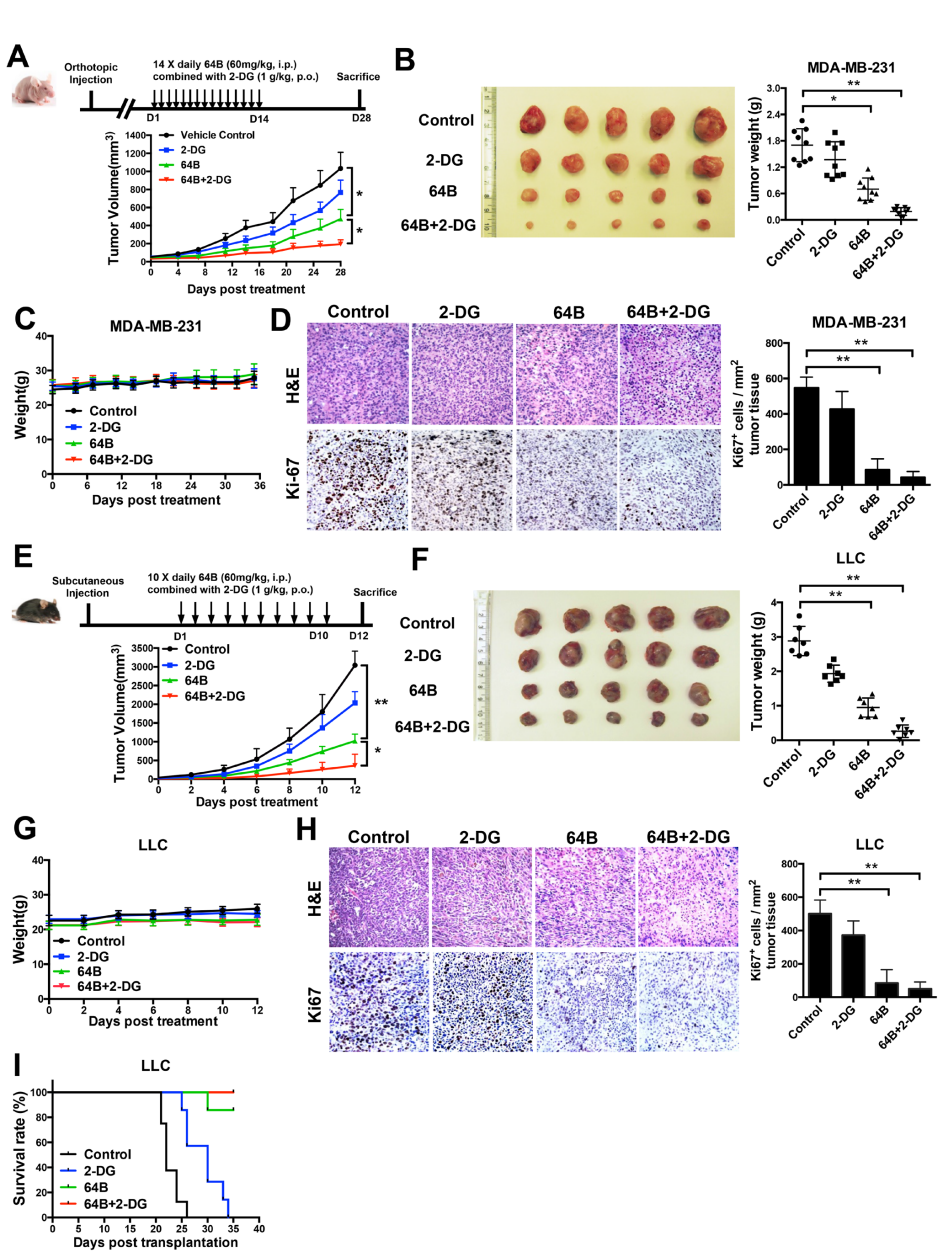
Combined treatment with 64B and 2-DG markedly retards tumor growth Mice received daily administration of 64B (60 mg/kg, i.p.) and/or 2-DG (1 g/kg, p.o.) for indicated duration (n = 5 mice/group). **A**, Treatment scheme (upper) and tumor growth curves (lower) in mice bearing orthotopic MDA-MB-231 breast tumors. The mice were treated for 14 days and sacrificed on day 28. **B**, Image and weight of MDA-MB-231 tumors harvested at termination. **C**, Average body weight of mice bearing MDA-MB-231 tumors during the entire course of the study. **D**, H&E staining and Ki-67 immunostaining of the MDA-MB-231 tumor sections. Images were acquired at 40× magnification. Quantification of Ki-67-positive cells was determined microscopically (mean cell number per field ± SD, 12 fields per tumor). **E**, Treatment scheme (upper) and tumor growth curves (lower) in mice bearing subcutaneous LLC tumors. The mice were treated for 10 days and sacrificed on day 12. F, Image and weight of LLC tumors harvested at termination. **G**, Average body weight of mice bearing LLC tumors during the entire course of the study. **H**, H&E staining and Ki-67 immunostaining of LLC tumor sections. Images were acquired at 40× magnification. Quantification of Ki-67-positive cells was determined microscopically (mean cell number per field ± SD, 12 fields per tumor). **I**, Kaplan-Meier survival curves in mice bearing subcutaneous LLC tumors. Mice were euthanized when the tumor size reached IACUC termination criteria.The results are reported as the mean ± SD (n = 5). *, *p* < 0.05. **, *p* < 0.01.

We then evaluated the anticancer efficacy of 64B in two tumor models. In mice bearing orthotopic MDA-MB-231 triple-negative human breast tumor xenografts, daily treatment of 2-DG at an oral dose of 1 g/kg for 14 consecutive days modestly slowed down tumor growth, whereas 64B as a single agent at a daily i.p. dose of 60 mg/kg significantly retarded tumor growth (Figure 5A). The combined treatment with 64B and 2-DG further enhanced the anticancer efficacy and nearly completely arrested tumor growth. The weight of tumors harvested on day 28 confirmed the same trend (Figure 5B). The body weight of mice remained steady during the entire 4-week duration (Figure 5C), indicating no overt treatment-associated toxicities. Immunohistochemistry staining of the tumor tissues for the cell proliferation marker Ki-67 revealed that tumor cells in the 64B/2-DG-treated mice had the lowest mitotic index, while the tumors in PBS- and 2-DG-treated mice were highly proliferative (Figure 5D). Similarly, 64B/2-DG combination resulted in superior tumor growth retardation in mice bearing subcutaneous LLC tumors, a highly aggressive murine lung tumor model (Figure 5E, 5F, 5G, 5H). Treatment with 64B alone or in combination with 2-DG clearly improved the survival of LLC tumor-bearing mice (Figure 5I). Taken together, these results demonstrate potent anticancer efficacy of 64B *in vivo*, which can be further improved by the combined treatment with 2-DG.

**Figure 6:**
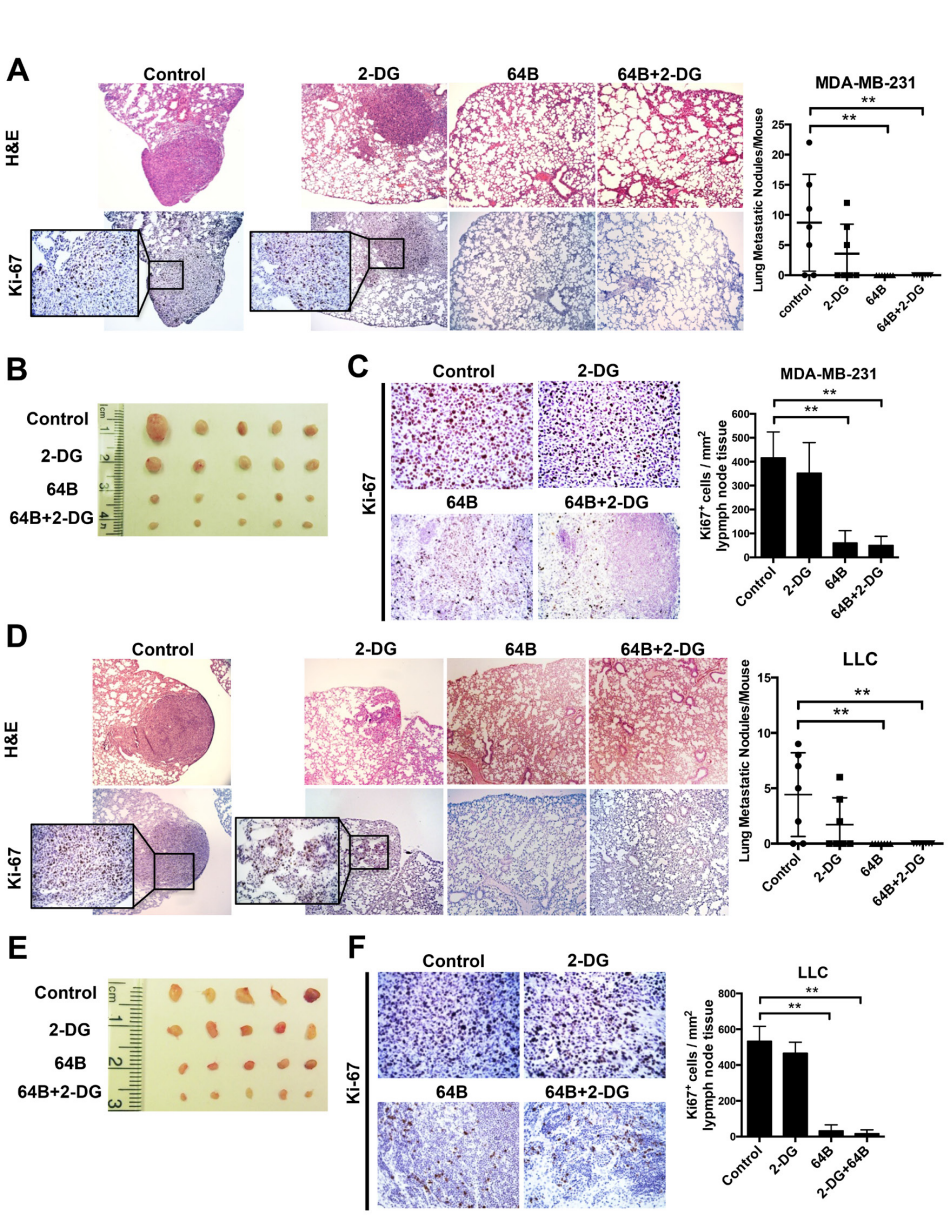
Combined treatment with 64B and 2-DG suppresses tumor metastasis **A**, H&E staining and Ki-67 immunostaining of the lung tissue sections in mice bearing orthotopic MDA-MB-231 tumors (sacrificed on day 28). Metastatic tumor nodules were counted in the five lobes of the lungs and the quantification is shown on the right. **B**, Images of the mesenteric lymph nodes harvested from mice bearing orthotopic MDA-MB-231 breast tumors. **C**, H & E staining and Ki-67 immunostaining of the mesenteric lymph node tissue sections in mice bearing orthotopic MDA-MB-231 tumors. Quantification of Ki-67-positive cells was determined microscopically (mean cell number per field ± SD, 12 fields per lymph node) and is shown on the right. **D**, H & E staining and Ki-67 immunostaining of the lung tissue sections in mice bearing subcutaneous LLC tumors (sacrificed on day 12). Metastatic tumor nodules were counted in the five lobes of the lungs and the quantification is shown on the right. **E**, Images of mesenteric lymph nodes harvested in mice bearing subcutaneous LLC tumors. **F**, H & E staining and Ki-67 immunostaining of the mesenteric lymph node tissue sections in mice bearing subcutaneous LLC tumors. Quantification of Ki-67-positive cells was determined microscopically (mean cell number per field ± SD, 12 fields per lymph node) and is shown on the right. The data are reported as the mean ± SD (n = 5). *, *p* < 0.05. **, *p* < 0.01.

Finally, as metastasis is the major cause of death in patients, we determined the impact of 64B on tumor metastasis in these two tumor models, both of which are highly prone to lung metastasis [26–28]. Orthotopic MDA-MB-231 triple-negative breast cancer-bearing mice developed an average of 8 metastatic tumor nodules in the lung on day 28 post-tumor cell inoculation (Figure 6A). Treatment with 64B alone or in combination with 2-DG completely prevented tumor nodule formation in the lung, while 2-DG individually reduced the lung metastases by half. The mesenteric lymph nodes in PBS- and 2-DG-treated mice were noticeably enlarged due to their colonization by of proliferating tumor cells (Figure 6B). In contrast, those from 64B- and 64B/2-DG-treated mice maintained a normal morphology and showed over 75% reduction in Ki-67 positive cells (Figure 6C). Similarly, complete blockade of lung metastasis was observed in mice bearing subcutaneous LLC tumors that received 64B treatment individually or in combination with 2-DG (Figure 6D), and the mesenteric lymph nodes in these mice were much less colonized with proliferating tumor cells (Figure 6E, 6F). These results provide strong evidence that 64B suppresses tumor metastasis *in vivo*.

## DISCUSSION

Tumor cells undergo pronounced metabolic reprogramming to engender macromolecular building blocks that are essential for rapid tumor cell proliferation and growth. As a master regulator for the biosynthesis of proteins, lipids and nucleic acids, mTORC1 plays a central role in coordinating anabolic pathways that ultimately drive tumor growth. Two of the best characterized mTORC1 immediate targets, S6Ks and 4E-BPs, are not only critically involved in protein synthesis by increasing ribosomal biogenesis and mRNA translational initiation, but also regulate lipogenesis and nucleotide synthesis [8, 9]. mTORC1 regulates translation of the mRNA encoding HIF-1α, the α subunit of hypoxia-inducible transcription factor HIF-1. Hypoxia is emerging as a key microenvironmental factor in the regulation of tumor metastasis, largely through HIF activation. Numerous HIF-1 target genes mediate invasiveness and the metastatic cascade during tumor progression [14]. Elevated HIF-1α protein levels in solid tumor biopsies correlate with metastasis in patients [29, 30], and inactivation of HIF-1 reduces the metastatic potential of tumor cells in animal models [31, 32].

We have had a long-standing interest in HIF-1 signaling and reported previously the synthesis and characterization of a series of arylsulfonamide compounds that potently and selectively inhibit the HIF-1 pathway [15–20]. Among them, N-((2,2-dimethyl-2*H*-chromen-6-yl)methyl)-3,4-dimethoxy-N-phenylbenzenesulfonamide (abbreviated as KCN1) markedly inhibits tumor growth and HIF activity in mice with little toxicity towards normal cells and tissues [17]. Recently, we have synthesized and screened more analogs of KCN1, among which 64B is found to be more potent than KCN1 (IC_50_∼0.3 vs. 0.6 μM) in a HIF-dependent cell-based luciferase reporter assay [15]. In the present study, we investigated the anticancer activity and mechanism of 64B *in vitro* and *in vivo*. We show that 64B downregulates mTORC1/HIF-1 signaling in tumor cells under glucose-limiting conditions. Under normoglycemic cell culture conditions, 64B interferes with glucose metabolism in tumor cells and elicits energetic stress that triggers AMPK activation. In tumor cells, the vast majority of glucose influx is known to be biotransformed into lactate and excreted into the extracellular space; yet, some glucose flux to the TCA cycle remain indispensible because of its crucial role in generating metabolic intermediaries and intracellular ATP [33]. We demonstrate that 64B elevates lactate production in tumor cells while reducing the conversion of pyruvate to acetyl-CoA, which hinders TCA flux and ensuing ATP production. Further studies are needed to elucidate the molecular mechanism by which 64B disrupts of pyruvate flux into the TCA cycle.

The metabolic and energetic stress caused by 64B is further potentiated by co-treatment with 2-DG, a glucose analog and glycolysis inhibitor that has been in clinical trials for cancer treatment [34–36]. Synergistic anticancer activities have been reported when 2-DG is combined with small-molecule inhibitors targeting the TCA cycle or OXPHOS [37, 38]. By dual inhibition of glycolysis and mitochondrial respiration, more severe energetic stress can be achieved in tumor cells. Our *in vitro* studies clearly evidence that 64B is highly effective at inhibiting ATP production in cultured tumor cells under glucose-limited growth conditions or pharmacologically-induced glycolysis inhibition. Results of *in vivo* investigation further demonstrate that 64B possesses remarkable anticancer efficacy in animal models for cancer. 64B notably inhibits tumor growth when administered as a single agent or in combination with 2-DG in orthotopic MDA-MB-231 triple-negative breast cancer and subcutaneous LLC lung cancer mouse models, both of which are known for highly aggressive tumor growth and metastatic propensity to the lung. While having little efficacy as a monotherapy, 2-DG markedly augments tumor growth retardation by 64B, probably owing to a synergy in blocking tumor cell ATP production.

Most importantly, 64B exhibits strong anti-metastatic effect *in vivo*, completely blocking metastatic tumor nodule formation in the lung. The significant reduction in tumor burden at the primary site in 64B-treated mice likely minimizes tumor cell extravasation into the circulation. Whether 64B also impedes colonization in the distant organ remains to be established. EMT is an enabling step towards tumor metastasis, and is characterized by upregulation of mesenchymal markers such as intermediate filament protein vimentin and reduced expression of the normal epithelium adhesion protein E-cadherin [21]. Snail1 is a transcription factor that binds the promoter region of *CDH1*, encoding E-cadherin, to repress its transcription. Consistently, 64B-treated tumor cells display impaired migratory and invasive potential, which is accompanied by decreased protein levels of Snail1 and vimentin as well as an increase in E-cadherin expression. The translation of both *vimentin* and *Snail1* mRNAs, along with a number of other pro-invasion messenger RNAs, is controlled by mTORC1 [39, 40], likely explaining their inhibition and restoration of E-cadherin in 64B-treated tumor cells.

We demonstrate that the anticancer activity of 64B is inversely affected by the glucose content to which tumor cells are exposed, and glucose deprivation potentiates 64B activity. It has been previously reported that glucose level influences the cytotoxicity of metformin, a Complex I inhibitor that halts ATP production by disrupting electron transport during mitochondrial respiration [41, 42]. Our data lend further support to prior findings that elevated glucose levels negate therapeutic interventions that target tumor glucose metabolism. Although growth media such as DMEM with high glucose content (4.5 g/L, 25 mM) are routinely used to cultivate tumor cells *in vitro*, they induce a metabolic phenotype that is drastically different from the much less permissive growth environment found within solid tumors [43]. Our findings underscore the importance of employing physiologically relevant glucose levels in cell cultures when evaluating anticancer agents that exploit glucose metabolic vulnerabilities.

In summary, our findings demonstrate that 64B potently suppresses tumor growth and metastasis in mouse models of triple-negative breast cancer and lung cancer. 64B perturbs tumor metabolism resulting in depletion of intracellular ATP and attenuation in mTORC1-mediated translation of critical pro-tumorigenic proteins including HIF-1 and EMT mediators that are critical for tumor growth and metastasis. The anticancer efficacy of 64B is further potentiated by glucose deprivation with 2-DG, compounding the metabolic and energetic stress exerted on tumor cells. Altogether, these data support further development of arylsulfonamides as a novel class of anticancer agents targeting metabolic vulnerabilities in cancer, especially in combination with a glycolysis inhibitor.

## MATERIALS AND METHODS

### Cell lines and reagents

MCF-7, MDA-MB-231, A549, NCI-H1975, Lewis lung cancer (LLC), BxPC-3, Panc-1, SK-MEL-28, B16-F10, LN229, MRC-5 and MCF-10A were obtained from the American Type Culture Collection (ATCC, Manassas, VA). Unless otherwise indicated, MCF-7, MDA-MB-231, A549, Panc-1, and MRC-5 were grown in DMEM (Life Technologies, Grand Island, NY) with 5.5 mM glucose (normoglycemic medium). LLC, SK-MEL-28 and LN229 were grown in DMEM with 25 mM glucose (hyperglycemic medium). NIH-H1975 and BxPC-3 were grown in RPMI 1640 medium, which contains 10 mM glucose (Life Technologies). All these media were supplemented with 10% fetal bovine serum (FBS) and 1% penicillin/streptomycin (Life Technologies). MCF-10A were cultured in DMEM/F12 (25 mM glucose) supplemented with 5% FBS, 20 ng/mL epidermal growth factor (EGF) (R&D Systems, Minneapolis, MN), 10 μg/mL insulin, 0.5 mg/mL hydrocortisone, 100 ng/mL cholera toxin (Sigma, St. Louis, MO) and 1% penicillin/streptomycin. All cells were cultured at 37°C in a 5% CO_2_ incubator. For the hypoxia conditions, cells were kept under 1% O_2_ in a modular incubator chamber (Billups-Rethenberg, Del Mar, CA) at 37°C.

64B was synthesized and characterized as reported in our previous work [15]. For all *in vitro* studies described below, a stock solution of 64B in DMSO was used to dilute to the final concentrations in the culture medium with DMSO concentration no more than 0.5% (v/v).

### Cell proliferation assay

Cells were seeded at a density of 5,000 cells/well in 96-well plates and treated with 64B (1–100 μM), and their viability was determined after 72-h treatment. To study the effect of *Glut-1* knockdown on the cytotoxicity of 64B, cells were transfected with *Glut-1* siRNA for 24 h by Lipofectamine 2000 (Life Technologies) according to the manufacturer’s protocol, followed by treatment with 64B (1–100 μM) for 48 h. To study the combination effect of 64B and 2-DG, cells were treated with 64B (1–100 μM) in the presence of 2-DG (2 mM) for 72 h. Subsequently, cells were fixed with 1% glutaraldehyde, stained with 0.1% crystal violet, and the crystals dissolved in 10% acetic acid. The absorbance was quantified at 595 nm on a FLUOstar Omega plate reader (Cary, NC). The relative cell viability was calculated as the percentage of absorbance of the drug-treated *vs.* the untreated controls.

### Colony formation assay

Following pre-treatment with 64B (5 μM) for 24 h, cells were seeded in 12-well plates at a density of 150 cells/well, and were incubated without 64B for another 14 days. The adherent cell colonies (with a minimum of 50 cells per colony) were fixed with 1% glutaraldehyde, stained with 0.1% crystal violet, imaged, and counted using an inverted microscope (Olympus, Tokyo, Japan).

### Cell cycle analysis

In 6-well plates (1.0 × 10^6^ cells/well) cells were treated with 64B (5 μM) for 24 h, and then fixed in 70% ethanol at 4°C overnight, washed with cold PBS, re-suspended in a staining solution 0.1% Triton-X-100, 50 μg/ml propidium iodide (PI) and 1 mg/ml RNase A for 30 min at room temperature, and analyzed by a BD Accuri C6 Flow Cytometer System (San Jose, CA). The DNA content distribution was analyzed using the FlowJo 9.3.1 software (Tree Star, Ashland, OR).

### Apoptosis assay

In 6-well plates, tumor cells (1.0 × 10^6^ cells/well) were treated with 64B (5 μM) for 24 h and stained with Annexin V-FITC and PI (BD Biosciences), which were analyzed by flow cytometry.

### Measurement of metabolic intermediaries of glucose

Tumor cells (1.0 × 10^6^ cells/well) were seeded in 6-well plates containing DMEM with 5.5 mM or 25 mM glucose. After 24 h, the medium was replaced with 2 mL fresh medium containing 64B (5 μM) and the cells were incubated for up to 24 h. Cells were lysed, and intracellular metabolites were measured using commercial kits detecting ATP (Abcam, Cambridge, MA), pyruvate (Sigma-Aldrich), acetyl-CoA (PicoProbe, Abcam) and NAD^+^/NADH ratio (BioVision, Milpitas, CA), respectively, according to the manufacturers’ recommended procedures. To measure pyruvate production, cells were cultured in pyruvate-free medium. Levels of glucose and lactate in the conditioned medium were analyzed by using a glucose colorimetric assay kit (Sigma-Aldrich) and an EnzyChrom™ L-Lactate assay kit (BioAssay Systems, Hayward, CA), respectively.

### Western blot analysis

Tumor cells were lysed using RIPA buffer supplemented with protease and phosphatase inhibitors. Cell lysates containing 40 μg of total proteins were resolved in 12% SDS−PAGE gels, and transferred onto nitrocellulose membranes (GE Healthcare, Pittsburgh, PA). Blots were probed with antibodies targeting the following proteins: p-mTOR, mTOR, p-AMPKα (Thr172), AMPKα, p-S6K1 (Thr398), S6K1, p-4E-BP1 (Thr37/46), 4E-BP1, p-EIF4G (Thr1108) and EIF4G (Cell Signaling, Danvers, MA); Snail1 and β-actin (Santa Cruz, Dallas, TX); HIF-1α and HIF-1β (BD Biosciences) and Glut-1 (Abcam). All antibodies were used at 1:1000 dilution. Protein band intensities were quantified by densitometric analysis using ImageJ software (NIH, Bethesda, MD).

### Quantitative real-time PCR (qRT-PCR) analysis

Tumor cells were lysed in Trizol (Invitrogen) for total RNA extraction. The expression of mRNAs of *HIF-1α*, *Glut-1* and *VEGF* was analyzed by qRT-PCR as previously described [44]. The sequences of the primers are listed in Supplementary Table 1.

### Animal experiments

All animal procedures were performed in accordance with the Guide for the Care and Use of Laboratory Animals of the National Institutes of Health. The Institutional Animal Care and Use Committee at Mercer University approved the protocol (Approval Number: A1105007). For the orthotopic breast tumor model, MDA-MB-231 human cells (1 × 10^6^ in 0.1 mL PBS) were injected into the fourth mammary pad of 6-week old female athymic nude mice (*nu/nu*, Charles River, Wilmington, MA). When the tumor size reached 50–100 mm^3^, the mice were randomized into four treatment groups (n=5 mice/group): (A) PBS control; (B) 2-DG; (C) 64B; (D) 64B/2-DG combination. 2-DG was orally (p.o.) administered to the mice at 1 g/kg once daily, while 64B was dissolved in Cremophor RH40:PEG400:deionized water at 3:1:8 (v/v/v) and intraperitoneally (i.p.) injected into the mice at 60 mg/kg once daily. For the mice receiving the 64B/2-DG combination therapy, the oral dosing of 2-DG was given 30 min prior to the i.p. administration of 64B. The mice were treated for 14 consecutive days. The tumor size was measured with a caliper twice weekly along with the mouse body weight, and the tumor volume was calculated as 1/2 × length × width^2^. For the subcutaneous lung tumor model, LLC cells (5 × 10^5^ in 0.1 mL PBS) were injected into the axillary region of syngeneic 6 week-old male C57BL/6 mice (Charles River). When the tumors were palpable, the mice were randomized into four treatment groups (n=5) as described above.

To observe tumor metastasis to the lung and lymphatic tissues, the tumor xenografts, lungs and lymph nodes in the tumor-bearing mice were excised for immediate formalin fixation. Formalin-fixed and paraffin-embedded tissue specimens of 3- to 4-μm thickness were sectioned and stained with hematoxylin and eosin (H&E) and a Ki-67 antibody (Cell Signaling) following standard immunohistochemistry protocols.

### Statistical analysis

All data were presented as the mean ± SD. Data from different groups were compared using Student’s *t*-test. A *p*-value of less than 0.05 was considered statistically significant.

## SUPPLEMENTARY MATERIALS FIGURES AND TABLE



## Abbreviations

2-DG: 2-deoxy-D-glucose
64B: N-cyclobutyl-N-((2,2-dimethyl-2*H*-pyrano[3,2-*b*]pyridin-6-yl)methyl)-3,4-dimethoxybenzenesulfonamide
α-KG: α-ketoglutarate
AMPK: AMP-activated protein kinase
EMT: epithelial-to-mesenchymal transition
HIF-1: hypoxia-inducible factor 1
KCN1: N-((2,2-dimethyl-2*H*-chromen-6-yl)methyl)-3,4-dimethoxy-N-phenylbenzenesulfonamide
mTORC1: mammalian target of rapamycin complex 1
OAA: oxaloacetate
OXPHOS: oxidative phosphorylation
PI: propidium iodide
TCA: tricarboxylic acid
