# Supplementary material for: A novel small-molecule arylsulfonamide causes energetic stress and suppresses breast and lung tumor growth and metastasis
Source: Oncotarget. 2017 Oct 29;8(59):99245–60. doi: 10.18632/oncotarget.22104 (PMC5725089; doi:10.18632/oncotarget.22104)
Supplement: Supplementary file 1 [file oncotarget-08-99245-s001.pdf]

A novel small-molecule arylsulfonamide causes energetic stress and suppresses breast and lung tumor growth and metastasis

SUPPLEMENTARY MATERIALS

Supplementary Table 1: The primer sequences for qRT-PCR analysis.

Gene		Sequence	
HIF-1α	Forward	5’-ACCCTCTTCGTCGCTTCG-3’	
	Reverse	5’-GGCCATTTCTGTGTGTAAGCA-3’	
VEGF	Forward	5’-CCTTGCTGCTCTACCTCCAC-3’	
	Reverse	5’-CACACAGGATGGCTTGAAGA-3’	
Glut-1	Forward	5’-TCCACGAGCATCTTCGAGA-3’	
	Reverse	5’-ATACTGGAAGCACATGCCC-3’	
β-actin	Forward	5’-ACAACGGCTCCGGCATGTGCAA-3’	
	Reverse	5’-CGGTTGGCCTTGGGGTTCAG-3’	

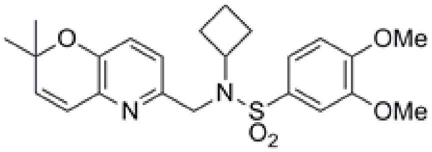

Supplementary Figure 1: Chemical structure of 64B.

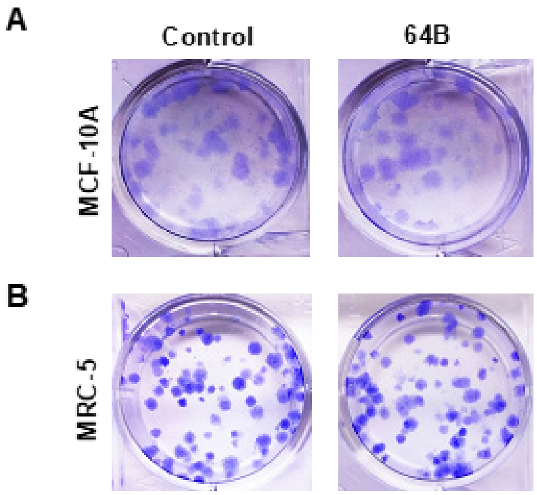

Supplementary Figure 2: Effect of 64B on colony formation of non-cancerous breast epithelial MCF-10A (A) and lung fibroblast MRC-5 cells (B) was visualized by crystal violet staining following 14-day incubation. Cells were pretreated with 5 μM 64B for 24 h prior to starting the assay. All data show representative results obtained from three independent experiments (n=3).

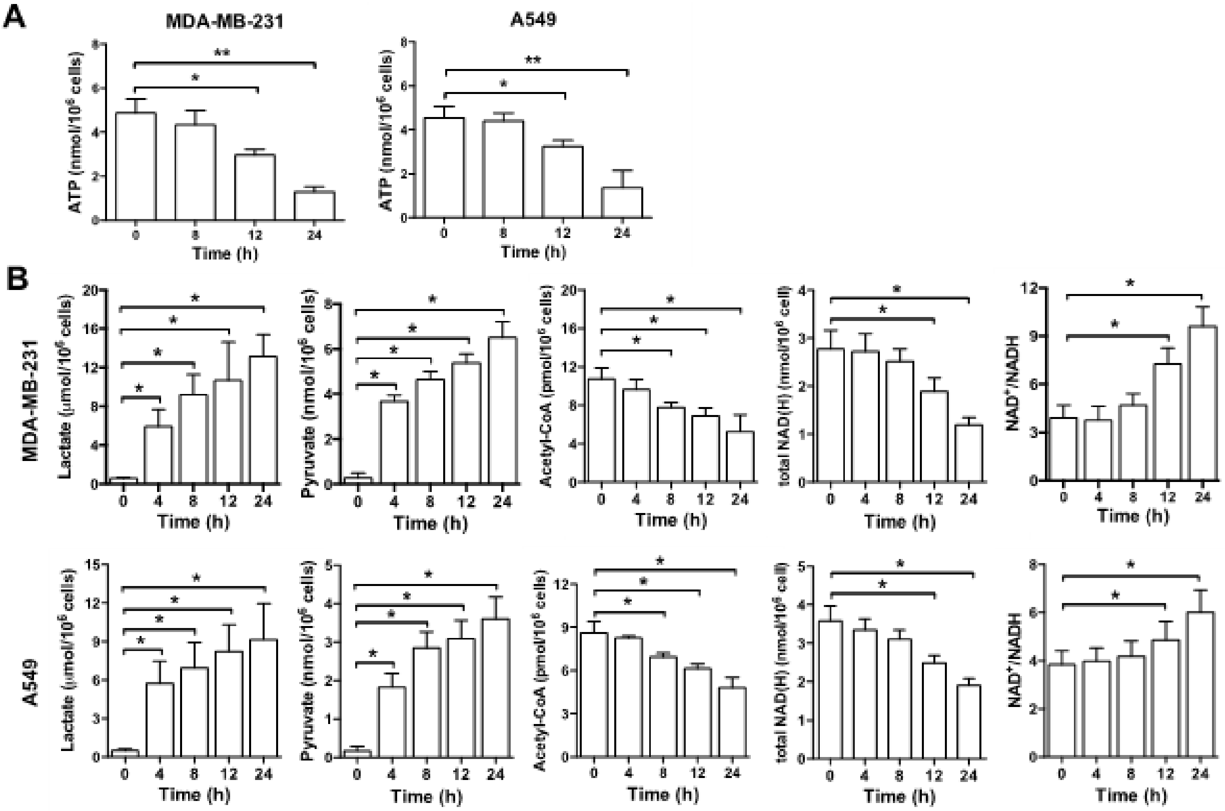

Supplementary Figure 3: Time course of ATP level (A) and metabolic intermediaries by measuring the levels of lactate, pyruvate, acetyl-CoA, total NAD(H), and the ratio of NAD+/NADH (B). MDA-MB-231 and A549 cells cultured in normoglycemic DMEM were treated with 5 μM 64B for up to 24 h. All data show representative results obtained from three independent experiments and the results are reported as the mean ± SD (n = 5). *, $p < 0.05$. **, $p < 0.01$.

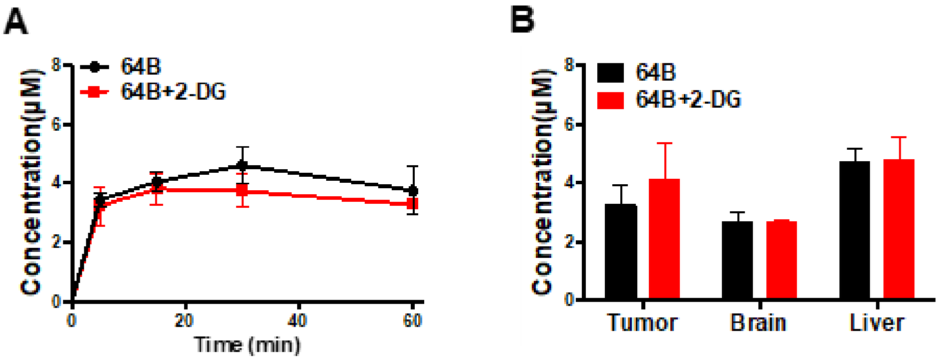

Supplementary Figure 4: The plasma concentration-time curves for 64B in mice bearing orthotopic MDA-MB-231 breast tumor xenografts (A) and the distribution of 64B in tumor, brain and liver (B) following the administration of 64B (60 mg/kg, i.p.) as a single agent or in combination with 2-DG (1 g/kg, p.o.). The results are reported as the mean ± SD (n = 3).
